# Renal Monitoring in Older Adults Prescribed Lithium: Adherence to NICE/BNF Standards in an Older People’s CMHT

**DOI:** 10.1192/bjo.2026.11836

**Published:** 2026-06-30

**Authors:** Sita Shah, Emin Erkal, Serife Yozgatli, Cansu Buyukulas, Vandana Menon

**Affiliations:** Cambridge and Peterborough NHS Foundation Trust, Cambridge, United Kingdom

## Abstract

**Aims:**

Lithium is a mood stabiliser primarily used for treatment and prophylaxis of bipolar disorder. Around 25% of people on lithium will experience renal impairment. There is little guidance around when to consult nephrology and psychiatry for consideration of discontinuing lithium. Prompt referral to nephrology can reduce mortality, decline in eGFR and enable initiation of renal replacement therapy.

We aimed to audit all older adults on lithium open to the older people’s community team at Cambridge and Peterborough foundation NHS trust to look at our adherence to standard renal monitoring.

**Methods:**

The BNF suggests lithium levels should be monitored 3 monthly if ≥ 65 years old. The BNF recommends avoiding lithium in severe (eGFR 15-29 ml/min) chronic kidney disease (CKD). We looked at whether advice had been sought from nephrology or psychiatry.

NICE recommends repeat urea and electrolytes within 2 weeks if eGFR <60ml/min, urine albumin:creatinine (A:CR) and a urine dipstick to check for haematuria if new decline in renal function. NICE recommends a minimum if 1 urine A:CR if moderate CKD (eGFR 30-59 ml/min), 2 urine A:CR if severe CKD and 4 urine A:CR for end stage renal failure (eGFR <15ml/min) per year. We only looked at renal investigations and monitoring for patients with eGFR <60 ml/min.

**Results:**

7/73 patients had lithium levels checked >3 monthly. 24/73 had eGFR 30-59 ml/min, 2/73 had eGFR 15-29 ml/min and 1 person had an eGFR <15ml/min. Of the 3 with eGFR <30 ml/min, 1 was referred to psychiatry and nephrology, 1 referred to nephrology and 1 referred to neither. 4/27 had a new decline in renal function but none had all of the initial investigations. 23/27 had CKD but only 6 met recommended NICE urine A:CR testing frequency.

**Conclusion:**

There is limited compliance with established standards. These audit results highlight the need for psychiatrists to work with General Practitioners (GP). We have updated laboratory lithium levels to reflect 0.4-0.8mmol/L for 65-80 years old and 0.4-0.7 mmol/L for >80 years old. Our guidelines for psychiatrists and General Practitioners have been updated to include investigations for new and established CKD and when to consult nephrology and psychiatry for discontinuation advice. However, we need a larger consensus to agree on a point at which renal function has deteriorated enough to trigger referrals to specialties to discuss discontinuation early so that patients have sufficient time to be involved in discussions.

